# A psychometric analysis of the Caring Assessment Tool version V

**DOI:** 10.1002/nop2.286

**Published:** 2019-04-21

**Authors:** Jenny Sim, Samuel Lapkin, Joanne Joyce, Rob Gordon, Conrad Kobel, Ritin Fernandez

**Affiliations:** ^1^ School of Nursing, Faculty of Science, Medicine & Health University of Wollongong Wollongong New South Wales Australia; ^2^ School of Nursing & Midwifery, College of Science, Health & Engineering La Trobe University Bundoora Victoria Australia; ^3^ Australian Health Services Research Institute, Sydney Business School, Faculty of Business University of Wollongong Wollongong New South Wales Australia; ^4^ Centre for Research in Nursing and Health St George Hospital Sydney New South Wales Australia

**Keywords:** caring, Caring Assessment Tool, empathy, factor analysis, instrument development, nurse–patient relations, nursing, nursing care

## Abstract

**Aim:**

The aim of this study was to examine the factor structure and construct validity of the Caring Assessment Tool version V (CAT‐V) for patients in Australian hospitals.

**Design:**

Secondary analysis of CAT‐V surveys from the Australian Nursing Outcomes Collaborative (AUSNOC) data set was used. The CAT was originally developed in the United States of America.

**Methods:**

The 27‐item CAT‐V was administered to patients prior to discharge from eight wards in three Australian hospitals in 2016. The psychometric properties of the CAT were evaluated using item analysis and exploratory factor analyses.

**Results:**

Item analysis of surveys from 476 participants showed high levels of perceived caring behaviours and actions. Exploratory factor analysis revealed a two‐factor structure consisting of: Nurse–patient communication; and Feeling *cared for*. The CAT‐V is a reliable and valid instrument for measuring patients’ perceptions of the attitudes and actions of nurses in Australia.

## 1 INTRODUCTION

Nursing is a caring profession. Nurses provide *care* and ensure that their patients are *cared for* (Chipman 1991). Definitions of nursing include the provision of care as their central tenet. The International Council of Nurses (ICN) (2002) defines nursing as an activity that “… encompasses autonomous and collaborative care of individuals of all ages, families, groups and communities, sick or well and in all settings.” The American Nurses Association (ANA) (2017) describes nursing as “… the protection, promotion and optimization of health and abilities, prevention of illness and injury, facilitation of healing, alleviation of suffering through the diagnosis and treatment of human response and advocacy in the care of individuals, families, groups, communities and populations.” The concepts of care and caring feature strongly in these definitions.

Despite the fact that caring is at the heart of nursing, only minimal attention is focused on evaluation of the caring components of nursing practice. Most attempts to evaluate nursing care are focused on the relationship between patient safety and nurse staffing (Heslop & Lu, 2014; Unruh & Zhang, 2012) and do not generally include measures of caring or person‐centred care (Maben, Morrow, Ball, Robert, & Griffiths, 2012; McCance, Telford, Wilson, MacLeod, & Dowd, 2011). The absence of data about caring or person‐centred care in nursing indicator sets such as the National Database of Nursing Quality Indicators or Collaborative Alliance for Nursing Outcomes (CALNOC) is evidence for this (CALNOC, 2017; Press Ganey, 2017). Studies that examine caring are usually cross‐sectional in design and focused on: evaluation of the patient–nurse relationship; the presence of person‐centred approaches to care; or patient satisfaction (Duffy, Brewer, & Weaver, 2014; Keeley, Wolf, Regul, & Jadwin, 2015; McCance, Slater, & McCormack, 2008).

There is a strong global commitment to improving health care and ensuring that the care provided by nurses is of the highest possible standard (McCance, Wilson, & Kornman, 2016). Recent reports into health system failures have highlighted how fragile the healthcare system can be and made recommendations for nurses to improve patient outcomes through focusing on the culture of caring and development of person‐centred approaches to care delivery (Francis, 2013; Garling, 2008). National regulation bodies and industrial associations promote a person‐centred approach to care with a specific focus on caring cultures (Australian College of Nursing, 2014; Australian Commission on Safety & Quality in Health Care, 2011). Practical international examples are seen in the Foundation of Nursing Studies (2017) resources for creating caring cultures and the resources developed by the Victorian Government (Australia) for implementing person‐centred services in care of hospitalized older people (Department of Health & Human Services, 2015).

There is only limited empirical research that examines links between improved patient outcomes and the presence of caring cultures (Feo & Kitson, 2016). Research that examines this phenomenon is usually related to person‐centred care. This is seen in the positive associations between person‐centred care and patient outcomes for people who have experienced an acute myocardial infarction (Meterko, Wright, Lin, Lowy, & Cleary, 2010) and haematology–oncology patients (Radwin, Cabral, & Wilkes, 2009). The patient–nurse relationship is less frequently studied, but seen as pivotal in examining the effectiveness of person‐centred cultures (Duffy et al., 2014).

There are several approaches used to examine patient–nurse relationships and the caring attitudes and actions of nurses from a patient's perspective. These include surveys, interviews, observation and the use of patient stories. Most research is survey‐based, and several different instruments have been developed. Most of these instruments are based on well‐established theoretical frameworks such as Watson's theories of human caring (e.g., Caring Behaviours Inventory [CBI], Caring Assessment Tool [CAT]), Swanson's theory of caring (e.g., Caring Assessment of Care Givers instrument) or a combination of different theories (e.g., Caring Dimensions Inventory). A discussion of the theoretical foundations of these instruments is beyond the scope of this paper. The most frequently used instruments for assessing caring behaviours and action of nurses from the patients’ perspective in acute care hospitals are the CBI and the CAT (Kuis, Hesselink, & Goossensen, 2014).

The CBI was originally developed by Wolf and colleagues in 1994 and assesses patient and nurse perceptions’ of caring using identical self‐report surveys with a six‐point Likert scale (Wolf, Giardino, Osborne, & Ambrose, 1994). The CBI was revised in 2006 to a 24‐item scale (CBI‐24) for both patient and nurse surveys (Wu, 2006). Several studies have used the CBI‐24 with appropriate reports of reliability and validity (Keeley et al., 2015; Papastavrou, Efstathiou, & Charalambous, 2011; Patiraki et al., 2012). A 6‐item CBI (CBI‐6) for use by patients has also been validated (Coulombe, Yeakel, Maljanian, & Bohannon, 2002) and used in several studies (Edvardsson et al., 2015; Edvardsson, Watt, & Pearce, 2017).

The CAT was originally developed by Duffy in 1990 as a 100‐item survey to assess patients’ perceptions of nurse caring behaviours (Duffy, 1990). The CAT has been iteratively revised (Duffy et al., 2014; Duffy, Hoskins, & Seifert, 2007) and is currently (CAT‐V) a unidimensional 27‐item survey. The CAT is supported by the Quality Caring Model© (Duffy & Hoskins, 2003) which combines multiple theories from multiple disciplines to help explore the nurse's relationship with the patient and the contribution that nursing attitudes and actions have on patient outcomes (Kim, 2016). The CAT is completed by patients using either a paper‐and‐pencil approach (Duffy & Brewer, 2011) or via electronic survey (Duffy, Kooken, Wolverton, & Weaver, 2012). Iterative versions of the CAT have had different numbers of items (100, 36 and 27) and different factor structures (between 8 and 1), and each version has reported appropriate reliability and validity (Duffy et al., 2014, 2007; O'Nan, Jenkins, Morgan, Adams, & Davis, 2014). However, all of the studies using the CAT have been undertaken in different population groups in the USA.

The CAT was chosen as the data collection instrument in this study because of its conceptual link with the Quality Caring Model© and the use of the Quality Caring Model© as the foundational model for evaluating nursing practice in over 40 hospitals in the USA (Duffy et al., 2012). In addition, the CAT had previously been used in an electronic format and this was an important factor in this study (Duffy et al., 2012). Once the decision to use the CAT in the Australian Nursing Outcomes (AUSNOC) data registry had been made, it became appropriate, given the differences between the healthcare systems in the USA and Australia, to test the construct validity of the CAT‐V in the Australian healthcare context. Therefore, the purpose of this study was to examine the factor structure and construct validity of the CAT‐V using exploratory factor analysis (EFA).

## 2 THE STUDY

### 2.1 Aim

The aim of this study was to examine the factor structure, reliability and construct validity of the CAT version V (CAT‐V) in the Australian healthcare setting using survey data collected in the AUSNOC data registry.

### 2.2 Design

The AUSNOC data registry is a multi‐site repository of structure, process and outcome measures that explore the quality and safety of nursing practice (Sim, Crookes, Walsh, & Halcomb, 2018). This study used cross‐sectional data from patients at the time of discharge in three hospitals who were participating in the feasibility testing of the AUSNOC data registry. The feasibility testing of the AUSNOC data registry is described elsewhere (Sim, Joyce‐McCoach, Gordon, & Kobel, 2019). Hospitals were chosen based on convenience and willingness to participate in the AUSNOC project. The data from the CAT‐V are focused on measuring patients’ perceptions of the caring attitudes and actions of nurses and the nurse–patient relationship.

### 2.3 Sample

Patients being discharged from three hospitals between March–December 2016 were approached to complete the CAT‐V survey. All hospitals included in this study were private hospitals providing acute care services in the state of New South Wales, Australia. Patients discharged from four surgical wards, three medical wards and one rehabilitation ward participated in the study.

### 2.4 Survey instrument

The CAT was originally developed in 1990 (Duffy, 1990) and is based on Watson's Theory of Human Caring (Watson, 2008). Several different versions of the CAT have been tested in hospitalized adults (Duffy & Brewer, 2011; Duffy et al., 2012; O'Nan et al., 2014), emergency department settings (Anosike, 2016), settings outside the USA (Melby, 2005), education settings to assess student relationship competency (CAT‐Edu) (Duffy, 2005) and among nurses to assess the caring behaviours of their managers (CAT‐Adm) (Wolverton, 2016). The most recent version of the CAT is referred to as CAT‐V and was validated by Duffy et al. (2014) for use with hospitalized adults. Table 1 provides an overview of the evolution of the CAT.

**Table 1:** Evolution of the Caring Assessment Tool (CAT) in published studies

CAT version	Characteristics	Psychometric properties
Original CAT (Duffy, 1990)	100‐item survey	Overall Cronbach *α* = 0.97
	8 factors	
CAT version IV (Duffy et al., 2007)	Validation study of original CAT	Cronbach *α* = 0.97 for original CAT
	Reduction to **36 items**	Cronbach *α* = 0.96 for CAT‐IV
	8 factors	Subscale coefficient *α* ranged from 0.76–0.92
CAT version V (Duffy et al., 2014)	Validation study of CAT‐IV	Cronbach *α* = 0.97
	Reduction to **27 items**	Single factor explained 73% of variance
	1 factor (unidimensional)	
CAT‐V (Current study, 2018)	Validation study of CAT‐V	Overall Cronbach *α* = 0.98
	27 items	Two factors explaining 72.44% of variance
	2 factors	Factor 1 Cronbach *α* = 0.97
		Factor 2 Cronbach *α* = 0.96

Bold items indicate revisions made to number of items in each iteration of the CAT's evolution.

The CAT‐V consists of 27 items and a single factor structure. Participants rate how often each item occurred in their healthcare experience on a five‐point Likert scale where 1 = never, 2 = rarely, 3 = occasionally, 4 = frequently and 5 = always. The CAT‐V includes items related to caring, person‐centred care and the nurse–patient relationship (Duffy et al., 2014). All items are directly related to the concept of caring which is defined by Duffy (2013) as “a process that involves the person of the nurse relating with the person of the patient” (p.32). No items in the CAT‐V are reverse scored. Summed scores for the overall scale range from 27–135, with higher scores indicating higher ratings of caring and person‐centred care (Duffy et al., 2014). In this research, pilot testing was undertaken using the CAT‐V with a sample of 40 patients from participating hospitals in February 2016. No changes were made to the wording of any items, and data from the pilot testing were not included in the final sample. Permission to use the CAT‐V was obtained under licence from QualiCare on 17/9/2015 (Licence #000915).

### 2.5 Ethical considerations

This study was approved by the Health and Medical Human Research Ethics Committee at the University of Wollongong and Illawarra Shoalhaven Local Health District (Approval No HE15/425). All participants were given a participant information sheet by a staff member in the ward and had the opportunity to ask questions about the study. Participants were free to choose whether they wanted to participate and provided informed consent prior to completing the survey. No identifiable data were collected from any participant. All data obtained in the survey were stored securely on password‐protected computer systems at the University of Wollongong.

### 2.6 Data collection

Participants completed the survey within 24 hr prior to discharge from the ward. Surveys were completed either by using an online survey tool in RedCap software (Harris et al., 2009) via an iPad™, or using a paper‐based form that was subsequently entered into the online survey tool by a nominated staff member in each ward. The survey consisted of demographic questions and the 27 item CAT‐V survey. All paper‐based forms were given a unique identifier, and data entry accuracy was verified in a random selection of surveys.

### 2.7 Data analysis

Prior to undertaking the psychometric analysis, missing value imputation and descriptive analyses were undertaken. The expectation–maximization technique was used to impute the missing values as it is reported to be the best method that produces unbiased estimates (Allison, 2012). Descriptive statistics were then used to summarize the demographic data. A two‐step approach involving both confirmatory factor analysis (CFA) and EFA adopted in previous studies (Bhagwat, Kelly, & Lambert, 2012; Servidio, 2017) was then used to examine the psychometric properties of the CAT‐V. The two‐step process is more feasible than a study replication in that the two‐step process enables researchers to run CFA and EFA independently on both samples to compare and confirm the results (Schumaker & Lomax, 2004). The data (*N* = 476) were randomly split into two subsamples of approximately 50% of the cases using the SAMPLE command in SPSS version 22.0 (IBM Corp, 2013). The first sample (*N* = 234) was used to test the fit of the unidimensional (one‐dimensional) model as per the original CAT‐V (Duffy et al., 2014) by CFA using a range of goodness‐of‐fit indices used in structural equation modelling. The second sample (*N* = 242) was used to derive the EFA to provide additional evidence of the psychometric strength of the CAT‐V. Face validity was also used to confirm whether the items in each factor were coherently related to each other in a manner consistent with the CAT‐V. Finally, Cronbach's alpha was calculated for the CAT‐V as an index of internal consistency. Generally, an acceptable alpha is >0.75 (Cronbach, 1951). All analyses were conducted using SPSS for Windows version 22 software and AMOS version 22 software (IBM Corp, 2013).

## 3 RESULTS

### 3.1 Demographics

A total of 2,103 patients completed surveys within the study period; however, examination of the data for completeness revealed 1,627 surveys with more than one item of missing data. This left 476 (22.63%) surveys included in the final sample for factor analysis. Most participants were female (*N* = 283, 59.45%). The most common age group was 60–79 years (*N* = 185, 38.87%), whilst approximately 10% were over 90 years old (*N* = 50, 10.50%). The participants were admitted under the following clinical specialities: Surgical (*N*: 266, 55.83%); Medical (*N* = 120, 25.28%); and Rehabilitation (*N*: 90, 18.89%). The length of stay ranged from 1 day–4 weeks.

### 3.2 Descriptive statistics

The means and standard deviations for each item in the CAT‐V (*N* = 476) are displayed in Table 2. The responses were negatively skewed with most participants responding either “Frequently” or “Always” on most items (mean = 4.52, *SD*: 0.71). The CAT‐V inter‐item correlation ranged between 0.44–0.81 demonstrating that most selected items measure related phenomena. The subsamples were similar with no significant differences in the mean scores for all the 27 CAT‐V items.

**Table 2:** Descriptive statistics of each item in the Caring Assessment Tool version V (*N* = 476)

Item (item number)	Mean	*SD*
Help me to believe in myself (1)	4.48	0.91
Make me feel as comfortable as possible (2)	4.78	0.61
Support me with my beliefs (3)	4.51	0.96
Pay attention to me when I am talking (4)	4.77	0.61
Help me see some good aspects of my situation (5)	4.59	0.79
Help me feel less worried (6)	4.63	0.78
Anticipate my needs (7)	4.56	0.76
Allow me to choose the best time to talk about my concerns (8)	4.48	0.90
Are concerned about how I view things (9)	4.42	0.99
Seem interested in me (10)	4.66	0.74
Respect me (11)	4.81	0.58
Are responsive to my family (12)	4.69	0.74
Acknowledge my inner feelings (13)	4.51	0.90
Help me understand how I am thinking about my illness (14)	4.54	0.93
Help me explore alternative ways of dealing with my health problem/s (15)	4.22	1.16
Ask me what I know about my illness (16)	4.09	1.22
Help me figure out questions to ask other health professionals (17)	4.08	1.27
Support my sense of hope (18)	4.42	0.97
Respect my need for privacy (19)	4.72	0.66
Ask me how I think my health care treatment is going (20)	4.36	1.04
Treat my body carefully (21)	4.72	0.68
Help me with my special routine needs for sleep (22)	4.52	0.96
Encourage my ability to go on with life (23)	4.47	1.03
Help me deal with my bad feelings (24)	4.30	1.17
Know what is important to me (25)	4.49	0.98
Talk openly to my family (26)	4.50	1.00
Show respect for those things that have meaning to me (27)	4.61	0.87
Overall mean	4.52	0.71

### 3.3 Confirmatory factor analysis

The goodness‐of‐fit statistics for the 27‐item unidimensional model as per the original CAT‐V using the first subsample (*N*: 234) indicated a poor fit: (*χ* ^2^ = 1 882.74, *df* = 324; *p* < 0.001); Goodness of fit statistic (GFI) = 0.59, Root Mean Square Error of Approximation (RMSEA) (90% confidence interval [CI] = 0.14 (0.14, 0.15); CFI = 0.75; and Standardised root mean square residual (SRMR) = 0.08 (Table 3). Brown (2012) asserts that the RMSEA should be ≤0.06 (and no >0.08); and suggests that GFI and Comparative fit index (CFI) values should be >0.90 with values closer to 1.00 indicating a better model fit. Revised models based on the review of the modification indices, the specification of multiple correlated error terms and allowing covariance between identified items, did not result in improved fit. These results suggested that the 27‐item unidimensional model was not the best fit for the data; hence, an EFA was conducted.

**Table 3:** Model fit indices of the CFA on the first subsample (*N* = 234)

Measure	Cut‐off criteria			Results	Interpretation
	Poor	Acceptable	Excellent		
CMIN/DF $\left(\chi^{2}/df\right)$	>5	>3	>1	5.81	Poor
GFI	<0.90	<0.90	>0.95	0.59	Poor
RMSEA	>0.08	>0.06	<0.06	0.14	Poor
CFI	<0.90	<0.90	>0.95	0.76	Poor
SRMR	>0.10	>0.08	<0.08	0.08	Excellent
RMSEA	>0.08	>0.06	<0.06	0.14	Poor
PClose	<0.01	<0.05	>0.05	0.00	Poor

### 3.4 Exploratory factor analysis (EFA)

The second sample (*N* = 242) was used to explore the dimensionality of the CAT‐V using EFA. Bartlett's test of sphericity revealed statistical significance (*χ* ^2^ = 7587.05, *df* = 351, *p* < 0.0001) indicating that the data were adequately distributed to allow an evaluation of the potential factor structure. The Kaiser‐Meyer‐Olkin (KMO) index was 0.961, suggesting that the ratio of the number of participants to CAT‐V items was sufficient for factor analysis.

Two factors had eigenvalues greater than one and accounted for 72.44% of the variance of the total factor loading. The inflexion on the scree plot and further analysis suggested a departure from linearity that was consistent with a two‐factor solution. Further attempts at different factor structures did not significantly change the number of residuals. Therefore, a two‐factor structure was considered best fit for these data. A summary of the EFA for the two subscales of the 27‐item CAT‐V is presented in Table 4. All items loaded 0.5 or higher on the respective factors. The two‐factor model was examined, and items thematically analysed to identify the relevant constructs. The first factor included 17 items with communalities ranging from 0.70–0.86 and described the nurse's engagement with their patient and presence during communication. Factor 1 was named “Nurse‐patient communication.” The second factor covered 10 items and explained the person's values, beliefs and their understanding of their illness/treatment. Factor 2 was named “Feeling cared for.” The communalities for the second factor ranged from 0.80–0.81. All authors met to examine the proposed structure and thematic analysis of the item names (and constructs being examined in these items). This process was used to identify an appropriate name for each factor.

**Table 4:** Rotated loading matrix of the exploratory factor analysis for the two‐factor Caring Assessment Tool version V solution (*N* = 242)

Item (item number)	Communalities	Two‐factor solution	
		Factor 1 loading	Factor 2 loading
Help me deal with my bad feelings (24)	0.86	**0.85**	0.29
Help me explore alternative ways of dealing with my health problem/s (15)	0.81	**0.84**	0.22
Help me figure out questions to ask other health professionals (17)	0.81	**0.82**	0.24
Ask me what I know about my illness (16)	0.80	**0.81**	0.25
Support my sense of hope (18)	0.88	**0.79**	0.42
Know what is important to me (25)	0.85	**0.79**	0.37
Encourage my ability to go on with life (23)	0.82	**0.75**	0.37
Help me understand how I am thinking about my illness (14)	0.87	**0.74**	0.50
Acknowledge my inner feelings (13)	0.86	**0.74**	0.49
Ask me how I think my health care treatment is going (20)	0.76	**0.72**	0.34
Are concerned about how I view things (9)	0.82	**0.70**	0.46
Allow me to choose the best time to talk about my concerns (8)	0.85	**0.69**	0.56
Talk openly to my family (26)	0.74	**0.66**	0.40
Support me with my beliefs (3)	0.79	**0.65**	0.52
Help me to believe in myself (1)	0.76	**0.62**	0.51
Show respect for those things that have meaning to me (27)	0.77	**0.59**	0.56
Help me with my special routine needs for sleep (22)	0.70	**0.56**	0.47
Respect me (11)	0.81	0.26	**0.88**
Make me feel as comfortable as possible (2)	0.85	0.23	**0.84**
Pay attention to me when I am talking (4)	0.78	0.30	**0.83**
Treat my body carefully (21)	0.81	0.31	**0.78**
Seem interested in me (10)	0.83	0.42	**0.76**
Respect my need for privacy (19)	0.83	0.37	**0.75**
Are responsive to my family (12)	0.86	0.44	**0.70**
Anticipate my needs (7)	0.77	0.51	**0.69**
Help me feel less worried (6)	0.79	0.61	**0.62**
Help me see some good aspects of my situation (5)	0.79	0.59	**0.61**
Explained variance (Total 72.44%)		*α* = 097	*α* = 0.96

Bold numbers indicate factor loadings.

### 3.5 Reliability and criterion‐related validity analysis

The Cronbach's alpha (*α*) reliability coefficient was 0.97 for “Nurse‐patient communication” and 0.96 for “Feeling cared for.” The overall internal reliability of the CAT‐V was *α* = 0.98. Acceptable internal consistency is usually indicated by a Cronbach's alpha of more than 0.70 (DeVellis, 2012). Therefore, these results suggest that the CAT‐V demonstrated high scale reliability. In addition, the two factors (“Nurse‐patient communication”: *M* = 4.41, *SD*: 0.84; “Feeling cared for”: *M* = 4.69, *SD*: 0.58) showed a high correlation of *r* = 0.83 (*p* < 0.001, two‐tailed), which supports the criterion‐related validity of the CAT‐V questionnaire.

## 4 DISCUSSION

The purpose of this study was to evaluate the psychometric properties of the CAT‐V in the Australian healthcare setting. The CAT‐V was assessed using (a) a pilot study with 40 participants; (b) analysis of data from 476 participants to establish a data set; and (c) a cross‐validation study to confirm the factor structure and to ensure reliability of the scale. Using CFA, the hypothesized unidimensional factor of the 27 item CAT‐V was rejected. The follow‐up EFA suggested a two‐factor model. Review of the items that loaded ≥0.50 on factor 1 led to the conceptual label “Nurse‐patient communication.” Revision of the items that loaded ≥0.60 on factor 2 led to the conceptual label “Feeling cared for.”

### 4.1 Reliability

Internal consistency of the CAT‐V was shown because the Cronbach's *α* confidence coefficient was higher than 0.75 (Cronbach, 1951) across the whole instrument and in each factor. The Cronbach alpha (*α*) values for the CAT‐V were 0.98. The Cronbach's alpha (*α*) for factor 1 (nurse–patient communication) was 0.97 and 0.96 for factor 2 (Feeling *cared for*). The high values of the alpha coefficients indicate that the instrument displays adequate internal consistency and therefore is a reliable measure for measuring the caring attitudes and actions of nurses as perceived by the person receiving care. The two‐factor solution which includes nurse communication and feeling cared for is consistent with the Quality Caring Model© (Duffy, 2013; Duffy & Hoskins, 2003).

### 4.2 Validity

The criterion‐related validity of the CAT‐V was supported by evidence of a high correlation between the two factors with *r* = 0.83 (*p* < 0.001, two‐tailed). Coefficients of 0.70 or higher are considered desirable (Polit & Beck, 2010). The construct validity of the CAT‐V was examined using EFA. The results of the EFA revealed a two‐factor model which assessed “Nurse‐patient communication” and “Feeling cared for.” Both concepts are seen as important in evaluating the caring attitudes and actions of nurses (O'Nan et al., 2014). The first factor “Nurse‐patient communication” consisted of 17 items, and the second factor “Feeling cared for” consisted of 10 items. Several items loaded on both factors (Item 5: Help me see some good aspects of my situation; Item 6: Help me feel less worried; Item 8: Allow me to choose the best time to talk about my concerns; and Item 27: Show respect for those things that have meaning to me). Each of these items was discussed by the research team, and the decision was made to leave them in the factor where they had the highest loading.

### 4.3 Development of the Caring Assessment Tool

Prior research has examined the factor structure of various versions of the CAT using EFA (Duffy et al., 2014, 2007). To the best of our knowledge, this is the first study to assess the factor structure of the CAT‐V; the first study to assess any version of the CAT in a data registry; and in the Australian context. Previous versions of the CAT have had a range of different subscales. The CAT‐IV had eight subscales (mutual problem‐solving; attentive reassurance; human respect; encouraging manner; appreciation of unique meanings; healing environment; affiliation needs; and basic human needs) (Duffy et al., 2007). The CAT‐V was reported as evaluating a unidimensional construct which was described as an expression of the nurse–patient relationship where the attitudes, skills and behaviours of nurses are assessed in the caring relationships they have with their patients (Duffy et al., 2014). The unidimensional CAT‐V described 73% of the variance in the construct and had a high Cronbach's alpha coefficient of 0.97 (Duffy et al., 2014). Our study has produced a two‐factor solution with an explained variance of 72.44% and a high Cronbach's alpha (*α*) coefficient of 0.98. This study builds on prior research and provides a valid instrument to advance the research in the field. This study has evaluated the psychometric properties of the CAT‐V and proposes a two‐factor solution in the Australian healthcare context. Data obtained for this study were obtained from multiple sites which enables generalizability of the results.

### 4.4 Study limitations

There are several limitations which must be considered when considering the results of this study. Firstly, a convenience sample from three hospitals in one state in Australia was used. As such, our results may not generalize to other locations. In addition, this study used self‐reported data which may limit the validity of findings as participants may have various reasons for over‐ or underestimating their responses due to social desirability and inaccurate recall. It is also possible that a substantial proportion of patients were not invited to participate in this study at time of discharge due to factors such as unexpected discharge, absence of key staff, busyness of the wards and staff not providing relevant information to potential participants at time of discharge. Despite these limitations, our findings make meaningful contributions to the body of knowledge and support the ongoing use of the CAT‐V to evaluating patients’ perceptions of the caring attitudes and actions of nurses at the time of discharge from an acute care hospital. Further evaluation of the CAT‐V with different types of patients and various age groups is required.

## 5 CONCLUSION

The results of this study support the usefulness of the 27‐item CAT‐V as a brief, reliable and psychometrically sound instrument for measuring patient's perceptions of the caring attitudes and actions of nurses. In evaluating the CAT‐V, a two‐factor structure was identified which highlights the ability to assess “Nurse‐patient communication” and “Feeling cared for.” The two‐factor, 27‐item CAT‐V provides important information at unit level about nurse caring that can be used to evaluate and improve the quality of nursing care provided to patients in hospitalized settings.

Assessment of nursing care quality is complex and multi‐faceted. In this study, the CAT‐V has been used to evaluate patients’ perceptions of the caring attitudes and actions of nurses during hospitalization. The CAT‐V provides important information about the quality of the patient–nurse relationship, communication and the perceptions of being cared for. These elements are essential to evaluate the quality and safety of nursing care in a holistic way (Sim et al., 2018). The two subscales of “Nurse‐patient communication” and “Feeling cared for” describe meaningful constructs that provide opportunities for hospitals to obtain more precise measures of the quality of nursing care. Additional studies that examine the factor structure of the CAT‐V and other measures of quality of nursing care are critically needed.

## CONFLICT OF INTEREST

None declared.

## AUTHOR CONTRIBUTIONS

JS, JJM, RG, CK: Study design. JS, SL, JJM, RG, CK, RF: Data collection and analysis. JS, SL, JJM, RG, CK, RF: Manuscript preparation.
